# β-Catenin-Associated Wnt Signaling and Tumor Microenvironment Markers in Basal Cell Carcinoma Subtypes

**DOI:** 10.3390/jcm15103804

**Published:** 2026-05-15

**Authors:** Tayfun Koçoğlu, Nilay Duman, Ahmet Çağrı Evran, Çiğdem Özdemir

**Affiliations:** 1 Private Dermatology Practice, Ankara 06680, Türkiye; 2 Department of Dermatology, Faculty of Medicine, Ege University, İzmir 35100, Türkiye; nilay.duman@ege.edu.tr; 3 Department of Medical Pathology, Edirne Sultan 1 Murat State Hospital, Edirne 22030, Türkiye; a_c_evran@hotmail.com; 4 Department of Medical Pathology, Afyonkarahisar Health Sciences University, Afyonkarahisar 03030, Türkiye; drcozdemir75@hotmail.com

**Keywords:** basal cell carcinoma, β-catenin, WNT3a, immunohistochemistry

## Abstract

**Background/Objective:** Basal cell carcinoma (BCC) is the most common cutaneous malignancy, arising from epidermal basal cells or the outer root sheath of the pilosebaceous unit. Despite its generally indolent clinical behavior, BCC exhibits substantial histopathological heterogeneity, which may reflect underlying biological differences among its subtypes. This study aimed to evaluate the expression of Wnt/β-catenin pathway components and tumor-associated markers—including COX-2, Ki-67, tryptase, CD1a, and WNT3A—across different histopathological subtypes of BCC. **Methods:** This retrospective cross-sectional study included 100 formalin-fixed paraffin-embedded (FFPE) BCC specimens retrieved between January 2006 and September 2015. After the exclusion of three cases due to inadequate tissue quality, the tumors were classified into nodular (*n* = 60), infiltrative (*n* = 16), superficial (*n* = 9), and other subtypes (*n* = 12). The immunohistochemical expressions of COX-2, Ki-67, CD1a, intratumoral and peritumoral tryptase, β-catenin, and WNT3A were assessed and compared among the BCC subtypes. **Results:** No significant differences were observed among the BCC subtypes regarding age or sex distribution. The expression levels of COX-2, Ki-67, CD1a, and mast cell-associated markers (intratumoral and peritumoral tryptase) did not differ significantly among the groups (all *p* > 0.05). Conversely, β-catenin expression was significantly higher in the infiltrative subtype compared with the other histological variants (*p* = 0.001). WNT3A immunoexpression was uniformly negative across all evaluated cases. **Conclusions:** Most of the evaluated immunohistochemical markers did not differentiate among the BCC subtypes. However, the significantly increased β-catenin expression observed in the infiltrative subtype suggests a potential association with tumor growth patterns rather than serving as a specific discriminative marker, thereby highlighting the biological heterogeneity of BCC. Although WNT3A expression was uniformly negative in all cases, this finding should be interpreted cautiously and does not allow for definitive conclusions regarding its role in Wnt pathway activation. Overall, these results support the need for further investigation into the Wnt/β-catenin pathway heterogeneity in BCC.

## 1. Introduction

Basal cell carcinoma (BCC) is the most common cutaneous malignancy in dermatopathology practice; however, its high incidence does not reflect biological uniformity [1,2]. Although most tumors exhibit indolent behavior with limited metastatic potential, a subset demonstrates infiltrative growth patterns, poorly defined borders, and increased local destructive capacity [3,4]. These observations support the concept that BCC represents a biologically heterogeneous disease influenced by histopathological subtype and tumor microenvironment rather than a single clinicopathological entity [1,2,3,4]. Histopathologically, BCC comprises distinct subtypes—including nodular, superficial, infiltrative, morpheaform/sclerosing, micronodular, and other variants—that differ in growth architecture, stromal response, and invasion characteristics [5,6,7]. Clinically, these differences are particularly relevant in high-risk anatomical sites such as the face and periocular region, where subtype directly influences surgical margin assessment and recurrence risk [7,8]. While nodular and superficial subtypes generally display more circumscribed growth patterns, infiltrative and morpheaform variants show irregular extensions into deeper dermal structures, contributing to incomplete excision and higher recurrence rates [2,5,8].

Although histopathological subtyping remains central to clinical decision-making, morphology alone may not fully capture tumor aggressiveness or underlying biological behavior [3,8]. Consequently, there is increasing interest in identifying immunohistochemical markers that can better characterize BCC biology beyond conventional histology. Among these, β-catenin, COX-2, Ki67, CD1a, and mast cell tryptase have been investigated as indicators of proliferative activity, inflammatory signaling, immune microenvironment, and tumor–stromal interactions [9,10,11,12,13,14].

The Wnt/β-catenin signaling pathway plays a key role in regulating cell proliferation, differentiation, and tissue homeostasis [15,16,17,18,19]. In its canonical form, β-catenin accumulates in the cytoplasm and translocates to the nucleus to regulate transcriptional programs involved in tumor progression and invasion [16,17,18,19,20]. Dysregulation of this pathway has been associated with enhanced proliferative capacity, altered cell adhesion, and invasive behavior in cancer biology [17,18,21,22], and may contribute to subtype-specific differences in BCC [9,10,11]. However, the mechanisms underlying pathway activation in BCC, particularly the contribution of specific Wnt ligands, remain incompletely defined.

In parallel, tumor microenvironmental components provide complementary insights into BCC biology. COX-2 links inflammation to tumorigenesis through the regulation of proliferation, angiogenesis, and apoptosis inhibition [14,23], while Ki67 reflects cellular proliferative activity, although its association with histopathological subtypes remains inconsistent [24,25,26,27,28,29,30,31]. CD1a-positive dendritic cells indicate immune surveillance and microenvironmental activity [32,33,34,35,36], and mast cell tryptase reflects stromal remodeling and angiogenic potential [37,38,39,40,41]. Taken together, these markers represent key biological processes—including proliferation, inflammation, immune response, and stromal interactions—that may interact with intracellular signaling pathways involved in tumor progression, including Wnt/β-catenin signaling. Despite increasing interest in these pathways, the extent to which they differ across BCC subtypes remains incompletely understood.

Therefore, the aim of this study was to evaluate the expression of Wnt/β-catenin signaling components alongside COX-2, Ki67, CD1a, and mast cell tryptase across different histopathological subtypes of BCC, in order to better characterize the biological basis of tumor heterogeneity.

## 2. Materials and Methods

### 2.1. Study Design and Patient Selection

This retrospective cross-sectional immunohistochemical study was conducted on archival formalin-fixed, paraffin-embedded (FFPE) tissue samples. Patients diagnosed with BCC at the Department of Dermatology and Venereal Diseases, Afyon Kocatepe University Faculty of Medicine, were included, with histopathological evaluation performed in the Department of Pathology. The study period extended from 1 January 2006 to 1 September 2015.

A total of 100 cases were initially identified; three were excluded due to inadequate paraffin block quality for immunohistochemical analysis, resulting in a final cohort of 97 patients. In cases with multiple lesions, one representative tumor per patient was included. Demographic data, including age and sex, were retrieved from medical records. The study was approved by the Institutional Ethics Committee and conducted in accordance with the Declaration of Helsinki. Informed consent was obtained from all participants.

All hematoxylin–eosin–stained slides were re-evaluated, and the most representative tumor section was selected for analysis. Histopathological subtypes were classified as nodular, superficial, and infiltrative, while less frequent variants (adenoid, micronodular, keratotic, pigmented, and pleomorphic) were grouped as “other” due to limited sample size.

#### 2.1.1. Immunohistochemistry

Immunohistochemical staining was performed on 4–6 µm FFPE sections using a biotin-free polymer-based detection system (Bond Polymer Refine Detection; Leica Biosystems, Newcastle, UK) on a Leica BOND-MAX automated platform (Leica Biosystems, Buffalo Grove, IL, USA). All antibodies were applied in a single run with appropriate positive and negative controls to minimize inter-assay variability.

The antibody panel included COX-2 (clone SP21, 1:100; Thermo Fisher Scientific, Waltham, MA, USA), Ki-67 (clone RB-9043, 1:300; Thermo Fisher Scientific, Waltham, MA, USA), CD1a (clone AB-5, 1:100; Thermo Fisher Scientific, Waltham, MA, USA), mast cell tryptase (clone AA1, 1:2000; Santa Cruz Biotechnology, Dallas, TX, USA), β-catenin (clone RB-9035, 1:300; Thermo Fisher Scientific, Waltham, MA, USA), and WNT3A (clone SC-28824, 1:20; Santa Cruz Biotechnology, Dallas, TX, USA). Antigen retrieval was performed using Bond Epitope Retrieval Solution 2 (ER2; Leica Biosystems, Buffalo Grove, IL, USA) for COX-2, Ki-67, CD1a, and WNT3A, and Bond Epitope Retrieval Solution 1 (ER1; Leica Biosystems, Buffalo Grove, IL, USA) for tryptase and β-catenin. This panel was selected to evaluate tumor proliferation, inflammatory signaling, immune microenvironment, and Wnt/β-catenin pathway activity.

Immunohistochemical evaluation was performed using marker-specific semi-quantitative scoring systems. β-catenin expression was assessed using the Remmele and Stegner immunoreactive score (IRS; range 0–12), which combines staining intensity and the percentage of positive cells and is widely used for markers with heterogeneous subcellular localization. COX-2 expression was evaluated using a 0–3 cytoplasmic intensity scale, and Ki67 using a 0–4 nuclear labeling index scale. Mast cell tryptase was quantified by counting intratumoral and peritumoral mast cells in 10 high-power fields (×40), and CD1a-positive cells were expressed as the mean number of Langerhans cells per 1000 tumor cells.

WNT3A expression was assessed using a simplified semi-quantitative scoring system (range 0–6), as staining was uniformly absent or minimal across all cases. This approach was adopted to capture limited variation while minimizing overestimation of non-specific background staining. All slides were evaluated independently by two observers blinded to clinical and histopathological data, and discrepancies were resolved by consensus. Despite these measures, the semi-quantitative nature of scoring introduces a degree of subjectivity and potential observer variability.

#### 2.1.2. Statistical Analysis

Statistical analyses were performed using IBM SPSS Statistics for Windows, Version 26.0 (IBM Corp., Armonk, NY, USA). Continuous and ordinal variables were analyzed using the Kruskal–Wallis test, while categorical variables were assessed using Pearson’s chi-square test or Fisher’s exact test, as appropriate. When overall significance was detected, post hoc pairwise comparisons were performed using the Mann–Whitney U test with Bonferroni correction. A *p*-value < 0.05 was considered statistically significant. The statistical approach was selected to account for non-normal data distribution, ordinal scoring systems, and unequal subgroup sizes inherent to retrospective histopathological studies.

## 3. Results

A total of 97 cases were included in the study. The mean age of the patients was 67.9 ± 12.7 years (range, 35–89 years). The cohort comprised 57 females (58.8%) and 40 males (41.2%). The demographic and histopathological characteristics of the cases are presented in Table 1.

COX-2 expression was generally low across the entire cohort, with 67% of cases showing no staining. Although COX-2 negativity appeared to be more frequent in the superficial subtype and relatively less frequent in the “other” subtype group, these differences were not statistically significant (*p* = 0.364). The distribution of COX-2 expression according to histological subtype is presented in Table 2.

**Figure 1:**
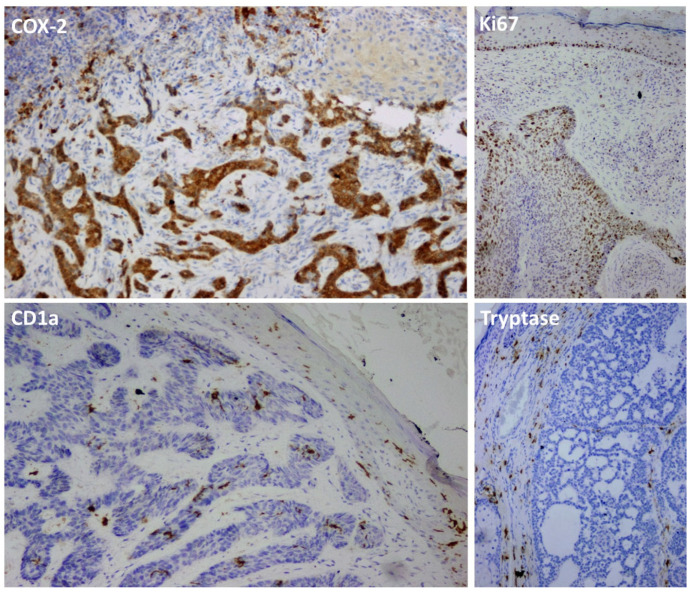
Representative immunohistochemical findings. Strong cytoplasmic COX-2 expression is observed in tumor cells and peritumoral inflammatory cells, with no expression detected in the adjacent squamous epithelium. The epidermis shows physiological basal nuclear staining, while the tumor exhibits a high proliferative Ki-67 index. Strong CD1a expression is evident in dendritic cells within both the epidermis and the tumor microenvironment. Cytoplasmic tryptase staining is present in peritumoral and intratumoral mast cells. Positive expression is visualized as brown staining (DAB), and nuclei are counterstained with hematoxylin (blue) (all images, ×200 magnification).

Similarly, no statistically significant differences were identified among the subtypes regarding the Ki-67 proliferation index (*p* = 0.696), CD1a-positive cell counts (*p* = 0.124), or intratumoral and peritumoral tryptase expression (*p* = 0.488 and *p* = 0.112, respectively) (Figure 1). Although statistically non-significant, CD1a expression was higher in the infiltrative and nodular subtypes compared to the other groups. Furthermore, peritumoral tryptase expression consistently exceeded intratumoral expression across all subtypes. The comparison of Ki-67, CD1a, and tryptase findings among the subtypes is summarized in Table 3.

**Figure 2:**
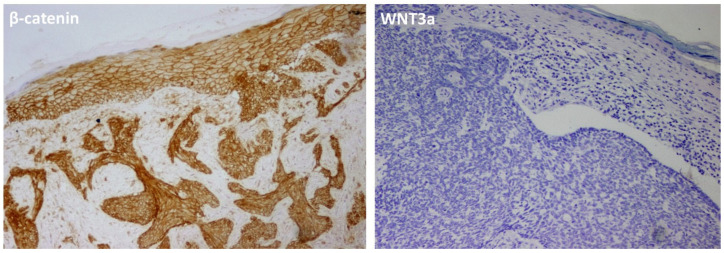
Representative immunohistochemical findings. Strong membranous and cytoplasmic β- catenin expression is observed in both the tumor cells and the overlying epidermis, whereas WNT3a expression is entirely absent. Positive staining is indicated by the brown chromogen (DAB), and background nuclei are stained with hematoxylin (blue) (all images, ×200 magnification).

In contrast, β-catenin expression demonstrated a statistically significant difference among the subtypes (*p* = 0.001). The highest expression levels were observed in the infiltrative subtype, whereas comparatively lower values were noted in the nodular and “other” subtype groups. The superficial subtype also displayed relatively elevated levels, albeit less pronounced than in the infiltrative group. Post hoc pairwise comparisons using the Mann–Whitney U test with Bonferroni correction revealed that β-catenin expression was significantly higher in the infiltrative subtype compared to the nodular subtype (*p* < 0.001), while no significant difference was observed between the nodular and superficial subtypes (*p* = 0.095). WNT3a expression was not detected in any of the examined cases (Figure 2). The evaluation of β-catenin and WNT3a expression according to histological subtype is presented in Table 4.

## 4. Discussion

This study evaluated the immunohistochemical landscape of BCC subtypes using a multi-marker panel integrating Wnt/β-catenin signaling, proliferation, inflammatory mediators, and tumor microenvironmental components. The main finding is that biological heterogeneity among BCC subtypes is not uniformly distributed across all markers but is selectively reflected, with β-catenin emerging as the only significantly differentially expressed marker, particularly in the infiltrative subtype. The demographic and clinicopathological features of the cohort were consistent with the literature, including the predominance of nodular BCC and the association with advanced age [1,2,5], while no significant differences were observed between subtypes in terms of age or sex.

The significantly higher β-catenin expression observed in infiltrative BCC supports a role of Wnt/β-catenin pathway dysregulation in tumors with distinct growth patterns. Given its dual function in cell–cell adhesion and transcriptional regulation, β-catenin likely reflects not only increased immunoreactivity but also alterations in tumor architecture and signaling dynamics. The concordance of our findings with previous reports describing enhanced β-catenin expression in more aggressive or infiltrative variants further supports this interpretation [10,11,12,13]. However, the presence of relatively elevated β-catenin expression in other subtypes, including superficial BCC, indicates that its role is not limited to tumor aggressiveness alone but may instead reflect differences in tumor architecture, differentiation status, and underlying signaling dynamics. This pattern suggests that Wnt/β-catenin pathway activation in BCC is context-dependent and varies across histopathological subtypes.

The coexistence of elevated β-catenin expression with uniformly negative WNT3A staining suggests that canonical ligand-mediated activation via WNT3A is unlikely to be the primary driver in BCC, and that pathway activation may instead arise from intracellular mechanisms, including stabilization of β-catenin or disruption of its degradation complex. However, the absence of WNT3A immunoreactivity should be interpreted with caution, as negative immunohistochemical findings do not necessarily indicate true absence of protein expression and may be influenced by technical factors such as antibody sensitivity, antigen retrieval conditions, or fixation-related epitope masking. Furthermore, this finding does not exclude the involvement of other Wnt ligands or receptors. Transcriptomic studies have demonstrated activation of Wnt signaling through alternative components, including WNT5A, WNT6, and Frizzled receptors [42], while WNT3 expression has been reported to be downregulated in nodular BCC compared with normal skin [42,43], supporting a context-dependent and heterogeneous role of Wnt ligands in this tumor type. In this context, the present findings should be interpreted as ligand-restricted observations rather than a comprehensive assessment of Wnt pathway activation in BCC. In contrast, WNT3A has been well characterized in other malignancies as an oncogenic ligand associated with increased proliferation and invasion [44,45,46,47], highlighting tumor-specific heterogeneity in Wnt ligand biology. Accordingly, the absence of WNT3A expression in our cohort may reflect pathway rewiring rather than classical ligand-dependent activation, and further studies incorporating broader molecular approaches, such as RNA-based or proteomic analyses, are warranted to clarify Wnt ligand involvement in BCC.

In contrast, COX-2, Ki67, CD1a, and mast cell tryptase did not demonstrate significant subtype-specific differences, suggesting that proliferation, inflammatory signaling, and microenvironmental immune components may represent general features of BCC rather than determinants of histopathological subtype. Although mast cell density was higher in the peritumoral area compared with the intratumoral compartment, this pattern did not differ across subtypes, supporting the presence of a reactive stromal microenvironment without subtype specificity. These findings indicate that subtype differentiation in BCC is unlikely to be driven by isolated changes in proliferation or inflammation alone, but rather by more complex architectural and signaling interactions.

A key strength of this study is the integrated evaluation of tumor cell–intrinsic and microenvironmental markers within a relatively large single-center cohort with standardized immunohistochemical processing. However, several limitations should be acknowledged. The retrospective, single-center design limits generalizability and introduces potential selection bias, while the extended inclusion period may have contributed to variability in tissue processing. The imbalance in subgroup sizes, particularly the relatively small number of superficial and rare subtypes, may have reduced statistical power, and the grouping of rare variants may have introduced additional heterogeneity. In addition, the semi-quantitative nature of immunohistochemical scoring may introduce observer variability despite standardized criteria. Finally, the absence of clinical outcome data, including recurrence, tumor size, anatomical localization, and treatment response, limits the ability to draw prognostic conclusions, and therefore the findings should be interpreted as descriptive rather than predictive.

## 5. Conclusions

This study demonstrates that BCC subtypes exhibit selective, rather than uniform, immunohistochemical differences. Variability in β-catenin expression across subtypes, with the highest levels observed in the infiltrative subtype, likely reflects differences in tumor growth patterns and underlying biological characteristics rather than aggressiveness alone. In contrast, COX-2, Ki67, CD1a, and mast cell tryptase did not show subtype-specific differences, and WNT3A expression was uniformly absent. Overall, these findings suggest that BCC biology may be driven by selective pathway alterations rather than widespread biomarker divergence, with β-catenin representing a context-dependent marker of tumor architecture. The absence of detectable WNT3A expression should be interpreted cautiously, and further studies are needed to clarify the role of Wnt pathway components in BCC.

## Figures and Tables

**Table 1:** Demographic and histopathological characteristics of basal cell carcinoma cases.

Subtype	*n* (%)	Age, Median (IQR)	Male, *n* (%)	Female, *n* (%)
Nodular BCC	60 (61.9)	66 (58–78.8)	24 (40.0)	36 (60.0)
Superficial BCC	9 (9.3)	73 (57–76)	3 (33.3)	6 (66.7)
Infiltrative BCC	16 (16.5)	74 (65–79.5)	8 (50.0)	8 (50.0)
Other subtypes *	12 (12.4)	73 (62.5–79.5)	5 (41.7)	7 (58.3)
*p*-value	—	0.286	0.855	—

Note: Data are presented as median (interquartile range, IQR) for continuous variables and as *n* (%) for categorical variables. Comparisons were performed using the Kruskal–Wallis test for continuous variables and Pearson’s chi-square test for categorical variables. * Other subtypes include adenoid, keratotic, micronodular, pleomorphic, and pigmented variants.

**Table 2:** Distribution of COX-2 expression in basal cell carcinoma subtypes.

Subtype	COX-2 Negative, *n* (%)	COX-2 Positive, *n* (%)	Total, *n*	*p*
Nodular	38 (63.3)	22 (36.7)	60	—
Superficial	8 (88.9)	1 (11.1)	9	—
Infiltrative	12 (75.0)	4 (25.0)	16	—
Other	7 (58.3)	5 (41.7)	12	—
Total	65 (67.0)	32 (33.0)	97	0.364

Note: Data are presented as numbers (percentages). Categorical variables were compared using Pearson’s chi-square or Fisher’s exact test, as appropriate. A *p* value < 0.05 was considered statistically significant.

**Table 3:** Comparison of Ki67, CD1a, and tryptase values among basal cell carcinoma subtypes.

Marker	Nodular (*n* = 60) Median (IQR)	Superficial (*n* = 9) Median (IQR)	Infiltrative (*n* = 16) Median (IQR)	Other (*n* = 12) Median (IQR)	*p*
Ki67 score	2 (1–2)	2 (1–3)	2 (1–2)	2 (2–2)	0.696
CD1a-positive cell count	7.5 (5–10)	5 (3.5–7)	7 (4–12)	6 (2–7)	0.124
Intratumoral tryptase-positive cell count	70 (82–130)	100 (72–160)	113 (92.5–140)	88 (71–145.5)	0.488
Peritumoral tryptase-positive cell count	151 (130–190)	180 (135–225)	150 (112–188)	123 (102.5–158.5)	0.112

Note: Data are presented as median (IQR). Comparisons among groups were performed using the Kruskal–Wallis test. A *p* value < 0.05 was considered statistically significant.

**Table 4:** β-Catenin and WNT3a expression in basal cell carcinoma subtypes.

Marker	Nodular (*n* = 60) Median (IQR)	Superficial (*n* = 9) Median (IQR)	Infiltrative (*n* = 16) Median (IQR)	Other (*n* = 12) Median (IQR)	*p*
β-Catenin score	2 (1–5.5)	3 (2.5–8)	**7.5 (4–12)**	0.5 (0–4)	**0.001**
WNT3a score	0 (0–0)	0 (0–0)	0 (0–0)	0 (0–0)	—

Note: Data are presented as median (IQR). Comparisons among groups were performed using the Kruskal–Wallis test. A *p* value < 0.05 was considered statistically significant. Bold values denote statistical significance.

## Data Availability

The data that support the findings of this study are not publicly available due to ethical and privacy restrictions but are available from the corresponding author upon reasonable request.
